# The influence of short-term complications on the outcomes of total elbow arthroplasty

**DOI:** 10.1016/j.jseint.2021.02.015

**Published:** 2021-04-16

**Authors:** Arno A. Macken, Ante Prkić, Niels Vermeulen, Iris van Oost, Koen L.M. Koenraadt, Bertram The, Denise Eygendaal

**Affiliations:** a Department of Orthopedic Surgery, Amphia Hospital, RK Breda, The Netherlands; b Foundation for Orthopedic Research, Care & Education, Amphia Hospital, RK Breda, The Netherlands; c Department of Orthopedic Surgery, Amsterdam University Medical Centres, DD Amsterdam, The Netherlands

**Keywords:** Total elbow arthroplasty, Complication, Ulnar nerve symptoms, Outcome, Satisfaction, Patient-reported outcome measures

## Abstract

**Background:**

The reported complication rate after total elbow arthroplasty is high, and objective outcomes are not always predictive of satisfaction. This study aims to investigate the effect of a short-term complication on patient satisfaction and patient-reported outcome measures.

**Methods:**

We retrospectively included 126 patients who received a primary total elbow arthroplasty at our hospital between 2008 and 2018 and compared outcomes between patients with a complication and patients without complications occurring within 1 year using t-tests or Mann-Whitney U tests. *P*-values were corrected using the Benjamini-Hochberg procedure.

**Results:**

In total, 26 patients developed a complication (21%). At the 1-year follow-up, there were no significant differences between the groups. At the 3-year follow-up, patients with a complication had a lower median satisfaction score (8 vs. 10; *P* = .0288) and Oxford Elbow Score (27 vs. 43; *P* = .0048). At the 5-year follow-up, there were no differences between the groups. However, the number of patients who completed the 5-year follow-up is low (42 patients).

**Discussion:**

Complications occurred in 21% of patients undergoing total elbow arthroplasty and lead to a decrease in satisfaction and Oxford Elbow Score after 3 years.

Total elbow arthroplasty (TEA) is a relatively uncommon procedure, and the results are not comparable with those in hip and knee arthroplasty.^16^^,^^24^ Clinical outcomes of arthroplasty are traditionally assessed using objective outcomes, such as implant survival, reoperation rate, or length of stay. However, these outcome measures are not always predictive of patient satisfaction.^4^^,^^10^ Nonsurgical perioperative factors such as empathy, management of expectations, and pain relief are factors that influence postoperative satisfaction the most.^4^^,^^11^^,^^14^

In comparison with hip and knee arthroplasties, the reported complication rate after TEA is relatively high, ranging between 11% and 38%.^9^^,^^19^^,^^22^ The most common complications after TEA are (early) loosening, infection, ulnar nerve symptoms, periprosthetic fractures, and triceps insufficiency.^22^ Besides the effect on the parameters mentioned previously, it is rational to expect that such a complication heavily impacts the patient’s satisfaction and reported elbow functionality. To quantify the outcomes of TEA from a patient’s point of view, patient-reported outcome measures (PROMs) can be used in the form of questionnaires, performance scores, quality of life, pain, or patient satisfaction measured on visual or numerical scales. ^2^^,^^5^^,^^20^^,^^23^ The current literature is sparse regarding the influence of a complication on the patient-reported outcomes or satisfaction after TEA. This study aims to investigate the effect of a short-term complication on patient satisfaction and PROMs.

## Materials and methods

All patients who underwent TEA at our hospital between 2008 and 2018 were identified. Exclusion criteria were revision surgeries of the implant with primary arthroplasty performed at an outside hospital, lack of completed follow-up visits or questionnaires, and a follow-up of less than 1-year. Implant types other than Coonrad-Morrey (Zimmer, Biomet, USA) were excluded to increase the internal validity of the study. This resulted in a cohort of 126 patients. Patients were included in the complication group if a complication occurred within 1 year after primary surgery. In case of revision surgery in which at least 1 component of the implant is replaced, the patient was censored at the time of revision. In case of bilateral elbow arthroplasty, only the implant that was placed first was included to minimize the chance of bias owing to previous experiences. Demographic data, surgical data, arc of motion, and complications, including nerve symptoms, triceps insufficiency, infection, and loosening or fractures, were extracted from the electronic patient files. Complications were registered in the electronic patient files as defined in the guideline of the Dutch Orthopedic Society including all adverse events that require a change of policy or cause transient or permanent impairment to the patient.^13^

PROM data were extracted from our digital follow-up system onlinePROMS (Interactive Studios, Rosmalen, the Netherlands).^20^ If patients had no access to the Internet or were not able to fill in the questionnaires online, the questionnaires were performed handwritten on paper and later added to the onlinePROMS system by the researchers. The collected outcome scores include the numerical rating scale for satisfaction ranging from 0 (completely unsatisfied) to 10 (completely satisfied); the EuroQol 5-dimensional questionnaire (EQ5D) combining all 5 dimensions (mobility, self-care, usual activities, pain/discomfort, and anxiety/depression) into a continuous score ranging from 0 (the worst outcome) to 1 (the best possible outcome); the Oxford Elbow Score (OES) in which 0 is the worst and 48 the best outcome; visual analog scales for pain in rest and pain during activities ranging from 0 (no pain) to 100 (the worst possible pain); and a visual analog scale for perceived health status with 0 being the worst perceived health status and 100 the best.

The patients were divided into 2 groups for the primary analysis: patients with a complication occurring within 1 year after primary surgery and patients without complications in the first year. First, the patient characteristics between the groups were compared using independent t-tests for continuous data in case of a normal distribution or Mann-Whitney U tests for skewed data; Fisher’s exact tests were used for categorical data, followed by post hoc t-tests per category in case of significance. For comparison between the complication and noncomplication groups with regard to outcome scores and arc of motion, independent t-tests were used for normally distributed data, and skewed data were analyzed using Mann-Whitney U tests. The Benjamini-Hochberg procedure was applied to both sets of analyses to correct for false positives. A corrected *P* < .05 was regarded as statistically significant. The data were analyzed using STATA software, version 14 (StataCorp, College Station, TX, USA), and R software (R Foundation for Statistical Computing, Vienna, Austria).

After approval of the institutional review board, 126 patients who received a TEA between 2008 and 2018 were included in this study. The mean age at the time of surgery was 70 years (standard deviation: 7.6), and the majority of the patients were women (83%). A posterior approach was used leaving the triceps intact in 40 patients (32%), dissecting the triceps in 85 patients (67%), and olecranon osteotomy was used in 4 patients (1%). The demographic data are described in Table I. At the time of the study, 126 patients (100%) had reached the 1-year follow-up period, 94 patients (75%) the 3-year follow-up period, and 55 patients (44%) the 5-year follow-up and were invited for their respective follow-up visits. The response rate was 100% for the preoperative objective outcomes and PROMs, and 92% (116 patients), 89% (84 patients), and 76% (42 patients) for the 1-, 3-, and 5-year postoperative follow-up, respectively. All patients completed at least 1 of the follow-up periods. At the time of the study, twelve patients (9%) were deceased, 2 patients (2%) declined further follow-up visits, and the remaining patients did not respond. Two patients were censored because a component was replaced in revision surgery. The median completed follow-up period was 3 years (range: 1-5 years). The data completeness at the specified follow-up moments was 65% for the preoperative outcomes, and 63%, 69%, and 73% for the 1-, 3-, and 5-year follow-up, respectively (Table I).

**Table I:** Demographics.

Completed follow-up period in yr, median (IQR)	3 (1-5)
Age in yr, mean (SD)	69.5 (7.6)
Female sex, n (%)	105 (83)
Right side, n (%)	56 (44)
Diabetes, n (%)	11 (9)
Smoking, n (%)	17 (13)
Complication, n (%)	26 (21)
Superficial wound infection	2 (2)
Deep infection	2 (2)
Radial nerve dysfunction	2 (2)
Transient ulnar nerve symptoms	13 (10)
Sensory	12 (10)
Motor	2 (2)
Requiring decompression of ulnar nerve	1 (1)
Permanent ulnar nerve damage	2 (2)
Fissure ulna	3 (2)
Hematoma	1 (1)
Triceps weakness	1 (1)
Sepsis	1 (1)

*IQR*, interquartile range; *SD*, standard deviation.

## Results

In total, 26 patients developed a surgery-related complication (21%). Complications consisted of ulnar nerve symptoms, radial nerve dysfunction, fissure fracture, hematoma, marked triceps weakness, infection, and sepsis. One patient with ulnar nerve symptoms required surgical decompression. In 2 patients, a deep infection occurred; in the first patient, the infection occurred 4 months postoperatively and was treated with a single irrigation and debridement and intravenous vancomycin. The second patient with a deep infection, occurring at 11 months postoperatively, required irrigation and debridement 3 times and was treated with intravenous amoxicillin and clavulanic acid. The remaining complications were treated conservatively. Two patients underwent a revision 4 years after primary surgery: a broken bushing was replaced in both cases, the results after replacement were censored. No other complications occurred after more than 1 year. (Table I)

After correction using the Benjamini-Hochberg procedure, we found a significant difference in the indications for TEA between the complication and noncomplication groups (*P* = .044). Post hoc analysis of the indications for TEA showed more patients with osteoarthritis in the complication group (19% vs. 3%, *P* = .011) and two patients with osseous metastasis of a primary tumor in the complication group vs. zero in the group without a complication (7% vs. 0%, *P* = .045). However, both indications are rare (8 and 2 patients, respectively). Other patient characteristics were comparable between the 2 groups, and there were no significant differences in preoperative PROMs between the groups. (Table II)

**Table II:** Comparison of cohorts.

Complication	Yes (n = 26)	No (n = 100)	Test statistic	*P* value	Corrected *P* value
Patient characteristics					
Age, mean (SD)	68 (8.7)	70 (7.3)	1.251	.213∗	.521
Female seks, n (%)	21 (81)	84 (84)		.391§	.661
Right elbow, n (%)	15 (58)	41 (41)		.199§	.521
ASA, n (%)				.925§	.969
1	1 (4)	3 (3)			
2	15 (58)	55 (55)			
3	11 (42)	36 (36)			
BMI, median (IQR)	26 (24-30)	27 (23-29)	−0.214	.737†	.910
Diabetes, n (%)	1 (4)	10 (10)		.454§	.667
Rheumatoid arthritis, n (%)	9 (35)	30 (30)		.816§	.910
Anticoagulant use, n (%)	2 (8)	17 (17)		.361§	.662
Smoking, n (%)	6 (23)	11 (11)		.199§	.521
Indication, n (%)				**.006**§	**.044**
Rheumatoid arthritis	6 (23)	27 (27)		.805‡	
Post-traumatic	12 (46)	56 (56)		.284‡	
Osteoarthritis	5 (19)	3 (3)		**.011**‡	
Fracture	2 (8)	12 (12)		.732‡	
Tumor metastasis	2 (8)	0 (0)		**.045**‡	
Previous surgery, n (%)	16 (62)	54 (54)		.827§	.910
Completed follow-up period in yr, median (IQR)	3 (3-5)	3 (1-5)	−1.289	.197†	.521
Preoperative measurements					
Health status, median (IQR)	69 (42-71)	66 (55-75)	0.269	.788∗	.910
Pain in rest, median (IQR)	52 (36-71)	45 (21-68)	1.028	.304†	.608
Pain during activities, median (IQR)	81 (71-90)	89 (79-93)	−1.384	.166†	.521
OES, median (IQR)	30 (13-60)	16 (7-60)	1.933	.053†	.292
EQ5D, median (IQR)	0.25 (0.19-0.73)	0.61 (0.31-0.81)	1.173	.241†	.530
Degrees of flexion-extension, median (IQR)	90 (75-110)	90 (60-105)	0.521	.602†	.830
Degrees of pronation-supination, median (IQR)	130 (115-150)	140 (120-150)	−0.755	.450†	.667
Treatment characteristics					
Approach, n (%)				**.001**§	**.011**
Triceps on	2 (8)	38 (38)		**.002**‡	
Triceps off	24 (89)	61 (61)		**.01**‡	
Olecranon osteotomy	1 (4)	0 (0)		.214‡	
Ulnar nerve release	25 (96)	94 (94)		1.000§	1.000
Postoperative casting, n (%)	23 (88)	44 (44)		**<.0001**§	**<.0001**

*ASA*, American Society of Anesthesiologists physical status classification; *BMI*, body mass index; *EQ5D*, EuroQol 5 dimentions; *IQR*, interquartile range; *MEPS*, Mayo Elbow Performance Score; *OES*, Oxford Elbow Score; *SD*, standard deviation.

*P* value corrected using a Benjamini-Hochberg procedure.

Bold text indicates a statistically significant *P*-value (<.05).

∗ T-test.

† Mann-Whitney U test.

‡ Post hoc using Fisher's exact test.

§ Others: Fisher's exact test.

At the 1-year follow-up, there were no significant differences in the outcomes between the groups.

At the 3-year follow-up, the numerical rating scale for satisfaction was worse in patients with a complication, with a median of 8 (interquartile range [IQR]: 7-9) compared with 10 (IQR: 9-10) in patients without a complication (*P* = .0288). The OES was also worse in patients with a complication, with a median of 27 (IQR: 20-37) compared with 43 (IQR: 35-47) for patients without a complication (*P* = .0048).

At the 5-year follow-up, there were no significant differences observed in the outcomes between the groups. (Table III)

**Table III:** Outcome.

Outcome, median (IQR)	Complication	No complication	Z value	*P* value	Corrected *P* value
1 yr	n = 23	n = 93			
Satisfaction	10 (9-10)	10 (9-10)	−.309	.757	.855
Health status	64 (50-77)	79 (66-85)	1.972	**.0486**	.157
Pain in rest	4 (0-17)	5 (0-15)	−.031	.980	.980
Pain during activities	24 (2-52)	17 (4-40)	−.655	.512	.768
OES	30 (24-39)	38 (28-46)	1.896	.058	.157
EQ5D	0.69 (0.69-0.81)	0.81 (0.69-0.90)	1.118	.264	.528
Flexion-extension	118 (95-123)	120 (110-130)	1.891	.059	.157
Pronation-supination	140 (120-160)	140 (130-150)	.305	.761	.855
3 yr	n = 23	n = 61			
Satisfaction	8 (7-9)	10 (9-10)	3.037	**.0024**	**.0288**
Health status	61 (30-80)	80 (57-85)	2.249	**.0245**	.147
Pain in rest	17 (2-40)	5 (1-25)	−1.261	.207	.452
Pain during activities	33 (9-70)	16 (5-40)	−1.910	.056	.157
OES	27 (20-37)	43 (35-47)	3.748	**.0002**	**.0048**
EQ5D	0.78 (0.69-0.84)	0.82 (0.81-1)	2.436	**.0148**	.118
Flexion-extension	123 (105-140)	120 (105-130)	−.700	.480	.768
Pronation-supination	125 (100-140)	140 (130-160)	2.160	**.0308**	.147
5 yr	n = 10	n = 32			
Satisfaction	10 (10-10)	9.5 (8-10)	−1.576	.115	.276
Health status	67 (50-80)	70 (60-79)	.274	.784	.855
Pain in rest	20 (0-35)	7 (2-33)	.384	.701	.855
Pain during activities	20 (0-70)	34 (4-54)	.281	.779	.855
OES	32 (27-43)	36 (30-43)	.835	.404	.746
EQ5D	0.80 (0.69-0.84)	0.81 (0.78-0.86)	.695	.487	.768
Flexion-extension	120 (105-130)	120 (110-126)	−.028	.978	.980
Pronation-supination	140 (100-160)	140 (120-140)	−.564	.573	.809

*EQ5D*, EuroQol 5 dimensions; *IQR*, interquartile range; *MEPS*, Mayo Elbow Performance Score; *OES*, Oxford Elbow Score.

*P* value corrected using Benjamini-Hochberg procedure.

Bold text indicates a statistically significant *P*-value (<.05).

## Discussion

To our knowledge, this is the first study assessing the influence of a complication on the outcomes of TEA. This study shows a short-term complication rate of 21% after TEA, with ulnar nerve symptoms being the most common complication. The patients with a complication had worse outcomes compared with patients without a complication after 3 years reporting lower satisfaction and OES scores. The difference in OES scores exceeds the minimal clinically important difference (ΔOES > 8).^7^ At the 1-year and 5-year follow-up, there were no significant differences in outcomes.

Our results reflect the impact of complications on the patient’s experience after TEA. Notably, in none of the follow-up periods, there was a difference between the groups in pain scores during rest or activity, which is usually an important predictor of patient satisfaction after total joint arthroplasty.^3^^,^^18^ This shows that PROMs are required to assess more complex aspects of daily life, other than pain, that are compromised owing to a complication.^1^ Similarly, we found no difference between the groups in arc of motion. Our results demonstrate that, even when pain scores and arc of motion remain unaffected, a complication may impact the patient’s satisfaction and elbow function.

Our results demonstrate an impact of complications at the 3-year follow-up, despite the majority of the complications occurring immediately after surgery (radial and ulnar nerve symptoms, ulnar fissure fracture and marked triceps weakness; 85%) and all complications occurring within 1 year. Interestingly, the 1-year follow-up outcomes showed no significant difference. The reason for this discrepancy is unclear. A logical explanation would be a larger variance in outcomes after 1 year; a previous study in hip arthroplasty described differences in short-term follow-up between groups of “fast starters,” “slow starters,” and “late dippers” which gradually level out after a longer follow-up.^6^ However, in our cohort, the means of the interquartile ranges of each outcome, taken as a percentage of the maximum score, show no substantial difference in spread between the 1-year and 3-year follow-up (23% and 25%), suggesting that there is not a wider spread of outcomes after 1 year. Another explanation could be that the perceived burden of a complication increases over time, and patients initially disregard their symptoms, whereas, if the symptoms persist, patients experience more nuisances. It is possible that functional limitations occur in an early stage but that there is a delay in the patient’s experience. Furthermore, complications that are conventionally labeled as “minor” or “transient,” such as ulnar nerve symptoms, may appear resolved but still impact the patient’s daily activities and experience. However, larger studies are required to confirm this effect, and consultation with a neurologist would be required to objectify ulnar nerve symptoms. At the 5-year follow-up, we found no differences between the groups. This could be explained by the fact that most complications are surgery-related and occur early in the postoperative process, suggesting that after 5 years, the complications have been resolved and the outcome scores of both groups approximate each other. However the number of patients included in the 5-year follow-up is low, it is possible that with a larger cohort significant differences may be found. Furthermore, long-term complications such as implant loosening or material failure may severely impact the satisfaction and PROM scores. In our cohort, 2 patients required partial implant replacement and were censored after 4 years.

The complication rate in the present study (21%) is congruent with reported complication rates in previous studies; Welsink et al^22^ published complication rates from 70 studies ranging between 11% and 38%. Transient ulnar nerve symptoms were the most common complication in our cohort, occurring in 16 patients (59% of all complications). Despite the ulnar nerve being released during surgery in the majority of our cohort (94%), thirteen patients had transient ulnar symptoms; of which, 11 were sensory, 1 was motor, and 1 was both. One patient had an ulnar nerve palsy that was resolved by ulnar nerve decompression, and 2 patients had permanent ulnar nerve damage. Intraoperative ulnar nerve release was not correlated with complications (*P* = 1.000). However, despite ulnar nerve symptoms often being classified as “minor” and “self-limiting” complications by clinicians, their impact on the satisfaction and health status should not be underestimated. Similarly, a previous study assessing PROMs after elbow contracture release found ulnar neuropathy to be a predictor of worse Disability of the Arm, Shoulder, and Hand scores and less postoperative improvement in Disability of the Arm, Shoulder, and Hand scores.^8^ Semicircular casting was also correlated with complications (*P* < .001). The pressure of soft-tissue swelling and hematoma formation may attribute to postoperative compression of the ulnar nerve, ultimately leading to nerve palsy. It is possible that refraining from semicircular casting can decrease ulnar nerve–related complications. Furthermore, the surgical approach dissecting the triceps was correlated with complications (*P* < .001). The triceps-on approach does not require splitting or manipulation of the triceps muscle and is, therefore, less prone to postoperative bleeding.^12^^,^^17^ However, after surgery with the triceps-on approach, patients are not treated with postoperative casting. Therefore, these findings may be confounded. In previous literature, no differences between the approaches in complication rates are described, but larger studies are required to determine the independent effects of casting and surgical approach.^19^^,^^21^

The majority of complications was surgery-related and occurred early in the postoperative process; nerve palsy, fissure fractures, and marked triceps weakness account for 85% of complications. The remaining 15% of complications are infectious sequelae; of which, deep infections (2 patients) tend to be the worst for both patients and clinicians.^15^^,^^21^ However, our cohort is too small to analyze the specific impact of this challenging complication.

The results of this study must be interpreted in light of its limitations. First, owing to the rare occurrence of TEA, the cohort size is small. Consequently, we were unable to perform a regression model to determine the independent effects of explanatory variables. However, the study was conducted at a large center specialized in TEA and includes one of the largest cohorts available nationally. Second, in collecting data retrospectively, this study relies on the accuracy and completeness of the electronic medical charts and online PROM system. Despite the improvement in follow-up rates owing to the implementation of the online PROM system,^20^ not all patients respond to the online questionnaires, potentially leading to an overrepresentation of the most satisfied or dissatisfied patients. This is inherent to the study design. Third, the number of patients who completed the 5-year follow-up questionnaires is relatively low, leading to an underestimation of the differences between the groups. However, the medians are comparable between the groups, and the statistical results show no trend toward significance. Furthermore, the majority of the results are concentrated around the positive end of their respective spectrum, demonstrating satisfactory outcomes in both groups. Fourth, the majority of the complications occurred immediately after surgery. Consequently, we did not perform a subanalysis of the time until a complication occurred. Finally, we included patients with Coonrad-Morrey implants exclusively, while increasing the internal validity of the study, the results may not be directly applicable to other implant designs.

## Conclusions

Complications occur in 21% of patients undergoing TEA. A short-term complication may lead to a decrease in patient satisfaction and OES scores at the 3-year follow-up compared with patients without a complication. Larger prospective cohort studies are required to confirm long-term results. Complications comprise a severe burden on the patient and the healthcare system, and more research is required to further prevent complications after TEA.

## Disclaimers:

*Funding:* No funding was disclosed by the author(s).

*Conflicts of interest:* Denise Eygendaal reports consultancy for Lima corporates, has given paid presentations for A.O. and Stryker and received institutional support from Matthys, Zimmer-Biomet and Stryker.

The other authors, their immediate family, and any research foundation with which they are affiliated have not received any financial payments or other benefits from any commercial entity related to the subject of this article.
